# The effect of avian blood on *Leishmania* development in *Phlebotomus duboscqi*

**DOI:** 10.1186/1756-3305-6-254

**Published:** 2013-09-02

**Authors:** Katerina Pruzinova, Jan Votypka, Petr Volf

**Affiliations:** 1 Department of Parasitology, Faculty of Science, Charles University, Prague, Czech Republic

**Keywords:** Leishmaniases, Vectors, Bloodmeal digestion, Trypsin, Chicken blood

## Abstract

**Background:**

The development of pathogens transmitted by haematophagous invertebrate vectors is closely connected with the digestion of bloodmeals and is thus affected by midgut enzymatic activity. Some studies have demonstrated that avian blood inhibits *Leishmania major* infection in the Old World vector *Phlebotomus papatasi*; however, this effect has never been observed in the New World vectors of the genus *Lutzomyia* infected by other *Leishmania* species. Therefore, our study was focused on the effect of chicken blood on bloodmeal digestion and the development of *Leishmania major* in its natural vector *Phlebotomus duboscqi*, i.e. in a vector-parasite combination where the effect of blood is assumed. In addition, we tested the effect of avian blood on midgut trypsin activity and the influence of repeated feedings on the susceptibility of sand flies to *Leishmania* infection.

**Methods:**

*Phlebotomus duboscqi* females were infected by rabbit blood containing *L. major* and either before or after the infection fed on chickens or mice. The individual guts were checked microscopically for presence and localization of *Leishmania*, parasite numbers were detected by Q-PCR. In addition, midgut trypsin activity was studied.

**Results:**

Sand fly females fed on chicken blood had significantly lower midgut trypsin activity and delayed egg development compared to those fed on rabbits. On the other hand, there was no effect detected of avian blood on parasite development within the sand fly gut: similar infection rates and parasite loads were observed in *P. duboscqi* females infected by *L. major* and fed on chickens or mouse one or six days later. Similarly, previous blood feeding of sand flies on chickens or mice did not show any differences in subsequent *Leishmania* infections, and there was equal susceptibility of *P. duboscqi* to *L. major* infection during the first and second bloodmeals.

**Conclusion:**

In spite of the fact that avian blood affects trypsin activity and the oocyte development of sand flies, no effect of chicken blood was observed on the development of *L. major* in *P. duboscqi*. Our study unambiguously shows that sand fly feeding on avian hosts is not harmful to *Leishmania* parasites within the sand fly midgut.

## Background

Digenetic parasites of the genus *Leishmania* (Kinetoplastida: Trypanosomatidae) alternate between intracellular amastigotes in mammalian hosts and extracellular promastigotes in sand fly vectors (Diptera: Phlebotominae). In the sand fly vector, development is confined to the digestive tract and is closely connected with bloodmeal digestion (reviewed by [1,2]).

The source of the bloodmeal influences the digestion and fecundity of females [3-7]. Proteolytic activity in the midgut of haematophagous insects is activated by ingested proteins and the consequent rate of trypsin activity is correlated with the protein content in the bloodmeal [8,9]. Thus, the reproductive potential of sand fly females partly depends on the type of bloodmeal and amount of ingested nutrients [4].

Ingested blood affects not only the digestion and fecundity of sand flies but also can affect *Leishmania* development. Sand fly midgut proteases influence *Leishmania* development and are one of the obstacles that parasites must overcome to establish an infection in the midgut (reviewed by [1,2] ). Adler [10] first suggested that products of blood serum digestion destroy *Leishmania* parasites in the midguts of ‘noncompatible’ sand fly species. According to Schlein and Romano [11] and Borovsky and Schlein [12], a specific component of the trypsin-like activity prevents the survival of *L. donovani* in the ‘noncompatible’ vector *Phlebotomus papatasi* while the ability to modulate this factor enables *L. major* to survive in ‘compatible’ sand fly species. Pimenta et al. [13] described the susceptibility of *Leishmania* to midgut digestion in the ‘compatible’ vector *P. papatasi* as stage-specific: *L. major* amastigotes and fully transformed promastigotes were relatively resistant to *P. papatasi* proteolytic activity, whereas parasites within the amastigote-to-promastigote transition were highly susceptible being killed.

However, even in ‘compatible’ vectors the bloodmeal from different animals has been described as having different effects on *Leishmania*[14]. Schlein et al. [15] reported that *Leishmania* infection is inhibited in its natural vector *P. papatasi* if the sand fly females were fed on turkeys before or after the infection. According to the authors, the parasite reduction is caused by the digestive process and a relatively high DNAase level is induced by nucleated avian erythrocytes. On the other hand, Nieves and Pimenta [16] tested the effect of nine different sources of blood (human, dog, horse, opossum, rodent, chick, chicken, mouse and hamster) on the development of *L. braziliensis* and *L. amazonensis* in *Lutzomyia migonei.* The bloodmeal source influenced the infection rates of the females, but none of the bloodmeal types (including avian blood) eliminated *Leishmania* parasites. Similarly, Sant’Anna et al. [17] noted that chicken blood supports the development of *L. mexicana* in *Lutzomyia longipalpis*. Moreover, in late-stage infections they found similar numbers of metacyclic promastigotes in females infected via rabbit blood or chicken blood [17]. These findings raised the hypotheses that there might be a difference in the effect of avian blood between the New World vectors of the genus *Lutzomyia* and Old World vectors of the genus *Phlebotomus*.

Since descriptions of the effects of avian blood on sand fly digestion and *Leishmania* development are contradictory, we studied the effect of mammalian and avian blood on the trypsin activity and oocyte development of *P. duboscqi*. In parallel experiments we tested whether the digestion of avian blood is harmful to the development of *L. major* in its natural vector: first we repeated the experiments done by Schlein et al. [15] but included proper control groups. Then, to explain our results we compared the susceptibility of *P. duboscqi* to *L. major* infection acquired in the first or second bloodmeal and in 100% versus 5% blood.

## Methods

### Sand fly maintenance

The colony of *P. duboscqi* was maintained under standard conditions as previously described [18].

### Fluorometric measurements of trypsin activity

Trypsin has been reported to affect *Leishmania* infections in sand flies [11,12]. Therefore, we measured trypsin activities after feeding on avian blood. Midguts of *P. duboscqi* females fed on rabbits or chickens were dissected at 18, 24, 30, 48, and 72 hours post blood meal (PBM) and transferred to 1.5 ml Eppendorf tubes. Each sample contained 10 midguts in 100 μl of Tris-NaCl (0.1 M Tris, 150 mM NaCl, pH = 8.44). The samples were homogenised and trypsin activity was measured in 96-well plate by a fluorometric assay with the substrate Boc-Leu-Gly-Arg-AMC (Bachem). Aminomethylcoumarin (AMC) was excited at 355 nm and fluorescence of released AMC was measured at 460 nm by a fluorometer (Tecane infinite M200). Data were evaluated statistically using main effect ANOVA (in STATISTICA 6.1 and StatSoft software).

### Protein assays of sand fly midgut homogenates

In bloodsucking insects, levels of proteolytic activity are known to correspond to the quantity and quality of proteins ingested during the bloodmeal [8,9]. To explain differences in midgut trypsin activities present after feeding on different blood sources, we measured the protein content of *P. duboscsqi* females. Midguts of *P. duboscqi* females fed on rabbits or chickens were dissected 4 hours PBM and transferred to 1.5 ml Eppendorf tubes. Each sample contained 15 midguts in 250 μl of Tris-NaCl (0.1 M Tris, 150 mM NaCl, pH 7.8).The samples were homogenised and total amounts of midgut protein were quantified according to the Bradford [19] method adapted to 96-well plates. Ten μl of midgut homogenates were mixed with 200 μl of the Bio-Rad protein assay reagent diluted 5× in distilled, deionised water. Absorbance was measured in 96-well plate at 595 nm by the Tecan infinite M200. Bovine serum albumin (Sigma, concentration 1 to 10 μg/well) was used as a standard.

### Experimental infections of sand flies

The *Leishmania major* strain LV561 (LRC-L137; MHOM/IL/1967/Jericho-II), the same strain as used by Schlein et. al [15], was maintained at 23°C on Medium 199 (Sigma) supplemented with 10% foetal calf serum (Gibco), 1% BME vitamins (Sigma), 2% human urine and gentamicin (80 μg/ml).

Females of *P. duboscqi* were fed through a chick-skin membrane on heat-inactivated rabbit blood containing 10^6^ promastigotes per ml. If not stated otherwise 100% rabbit blood was used. Blood-engorged females were separated and maintained on 50% sucrose. Bloodfed females were always maintained at constant temperature (26°C) because it is known that ambient temperature affects the digestion and *Leishmania* development within sand flies [20]. At various intervals post-infection (PI) the individual guts were checked microscopically for the presence and localization of *Leishmania* promastigotes. Parasite loads were graded according to Myskova et al. [21] as light (< 100 parasites/gut), moderate (100–1000 parasites/gut), or heavy (> 1000 parasites/gut). Data were evaluated statistically by means of the χ^2^ test using S-PLUS 2000 software.

The number of *Leishmania* parasites in individual females was counted using Q-PCR as described previously [21,22]. Briefly, experimental females were stored at −20°C and total DNA extraction was performed with a High Pure PCR Template Preparation Kit (Roche) according to the manufacturer’s instructions. Q-PCR using *Leishmania*-specific primers (forward: 5′-CTTTTCTGGTCCTCCGGGTAGG-3′; reverse: 5′-CCACCCGGCCCTATTTTACACCAA-3′ [22]) was performed by the SYBR Green detection method (iQSYBER Green Supermix, Bio-Rad, Hercules, CA) in Bio-Rad iCycler & iQ Real-Time PCR systems. Statistical evaluation was performed by the Kruskal-Wallis test and Mann–Whitney U-test using STATISTICA 6.1.

### The effects of sand fly feeding on avian blood before and after infection

To evaluate the effect of avian blood on *Leishmania* infection we followed three different experimental feeding schemes (the first two done according to Schlein et al. [15]): (i) We evaluated the effect of chicken blood taken before infection. Sand fly females fed either on chickens or mice were given a chance to lay eggs in breeding pots and then, after oviposition, were fed an infective bloodmeal (nine days after the first bloodmeal). (ii) In the second scheme we evaluated the effect of avian blood on parasites already present in the gut: females infected with promastigotes in diluted (5%) blood were fed either on chickens or mice one day PI. The decreased amount of nutrients in the diluted blood resulted in the females having to feed again without laying eggs. (iii) In addition, we evaluated the effect of avian blood during the later phase of *Leishmania* infection: females infected with promastigotes in diluted (5% or 10%) blood were fed either on chickens or mice six days PI. Initial experiments showed that 10% blood resulted in higher infection rates, and therefore in repeated experiments we used only this blood concentration.

### A comparison of sand fly susceptibility to *L. major* during the first and second bloodmeal

One group of *P. duboscqi* females was fed first on non-infected mice, allowed to lay eggs in a breeding pot and then (9 days post first blood feeding) infected experimentally, while the control group (one week younger) was maintained without a bloodmeal until the experimental infection. Both groups were infected simultaneously with the same parasite culture.

### The effect of 5% or 100% blood in the infective bloodmeal on parasite establishment in the sand fly midgut

To explain the results of infections done using diluted (5%) blood (experiment (ii)), we compared the infection rates and intensities of infection after feeding on 5% or 100% blood. Females were infected with promastigotes in diluted or undiluted rabbit blood and checked on days 1 and 2 PI; by this time infected females had fed on chickens or mice in the previous experiment.

### Differences in the digestion of 10% and 100% blood

To explain the results of infections done using diluted (10%) blood (experiment (iii)), we compared the trypsin activity after feeding on 10% and 100% blood. Midguts of females fed on 10% or 100% rabbit blood through a chick-skin membrane were dissected at 24, 30, 48, and 72 hours PBM and transferred to 1.5 ml Eppendorf tubes. Each sample contained a mixture of 10 midguts in 100 μl of Tris-NaCl (0.1 M Tris, 150 mM NaCl, pH = 8.44). Trypsin activity was measured by the fluorometric method described above.

In both groups of females, the time of defecation was also measured. Thirty fully blood-fed females from both groups were individually placed in small glass vials, maintained at 26°C and checked twice daily under a binocular microscope for defecation.

### Ethical statement

Animals were maintained and handled in the animal facility of Charles University in Prague in accordance with institutional guidelines and Czech legislation (Act No. 246/1992 coll. on Protection of Animals against Cruelty in present statutes at large), which complies with all relevant European Union and international guidelines for experimental animals. All the experiments (including sand fly feeding) were approved by the Committee on the Ethics of Laboratory Experiments of the Charles University in Prague and were performed under the Certificate of Competency (Registration Number: CZU 246/1992, CZ 00177).

## Results

### The effect of blood source on midgut trypsin activity

**Figure 1:**
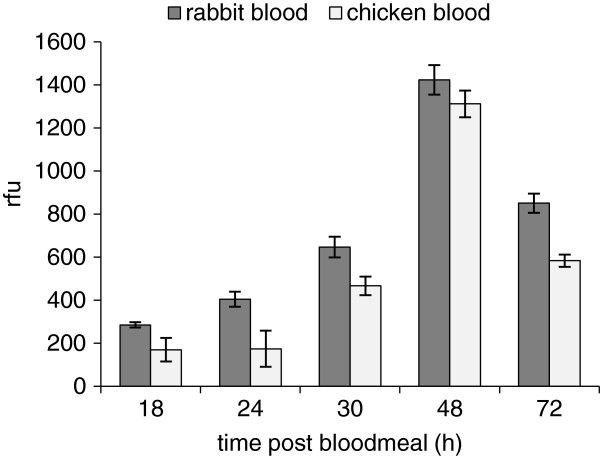
**Trypsin activity in the midguts of *P. duboscqi *females fed on rabbits or chickens.** Trypsin activity was measured at 18, 24, 30, 48, and 72 hours PBM in the homogenates of ten midguts of females fed on chickens or rabbits.

*Phlebotomus duboscqi* females were fed either on rabbits or chickens and dissected at 4, 18, 24, 30, 48, and 72 hours PBM, and protein absorbance and trypsin activity was measured. Data from two independent experiments were pooled. Sand flies fed on chickens had half the midgut protein content compared to those fed on rabbits (103 μg/gut versus 202 μg/gut, respectively). Similarly, midgut trypsin activity in females fed on chickens was significantly lower (F_(4,50)_ = 5.26, P < 0.01). The highest differences between groups were observed during the first 24 hours post bloodmeal, with females fed on chickens having 40 − 55% less trypsin activity in their midguts compared to those fed on rabbits (Figure 1).

### The effect of chicken and mice blood on *Leishmania* development in sand fly midguts

**Figure 2:**
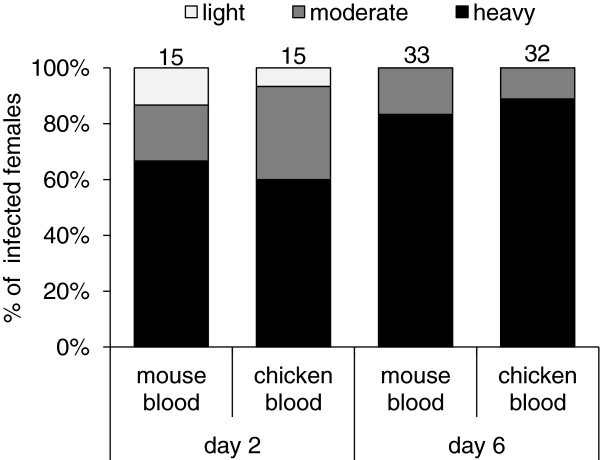
**The effect of chicken blood on *Leishmania *development in sand fly midguts: 9 days before infection.***P. duboscqi* females fed either on chickens or mice and nine days later were infected by 10^6^ promastigotes per ml of bloodmeal. Infection rates and intensities of infection were evaluated microscopically in sand fly midguts on days 2 and 6 PI, and infections were classified into three categories: light (< 100 parasites/gut), moderate (100–1000 parasites/gut), or heavy (> 1000 parasites/gut). Numbers above the bars indicate the number of dissected females.

**Figure 3:**
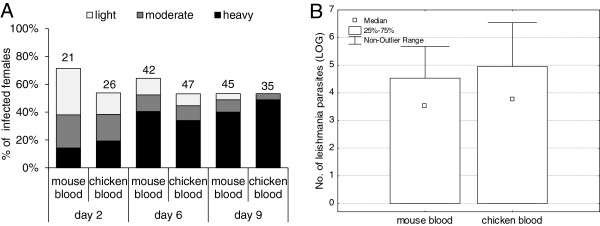
**The effect of chicken blood on *Leishmania *development in sand fly midguts: 1 day after infection.** One day after the infection by promastigotes in 5% rabbit blood (10^6^ parasites/ml) *P. duboscqi* females were allowed to feed on chickens or mice. Infection rates and intensities of infection were evaluated in sand fly midguts on days 2, 6, and 9 after the second bloodmeal. **A**: Intensities of infection were microscopically classified into three categories: light (< 100 parasites/gut), moderate (100–1000 parasites/gut), or heavy (> 1000 parasites/gut). Numbers above the bars indicate the number of dissected females. **B**: The precise number of parasites from 50 females of both groups was measured by Q-PCR 6 days after the second bloodmeal.

**Figure 4:**
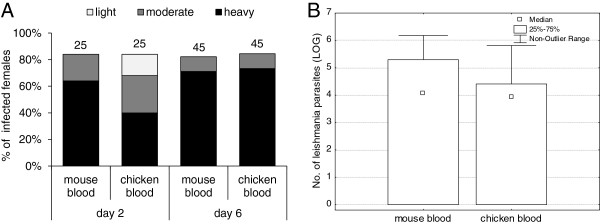
**The effect of chicken blood on *Leishmania *development in sand fly midguts: 6 days after infection.** Six days after the infection by promastigotes in 10% rabbit blood (10^6^ parasites/ml) *P. duboscqi* females were allowed to feed on chickens or mice. Infection rates and intensities of infection were evaluated in sand fly midguts on days 2 and 6 after the second bloodmeal. **A**: Intensities of infection were microscopically classified into three categories: light (< 100 parasites/gut), moderate (100–1000 parasites/gut), or heavy (> 1000 parasites/gut). Numbers above the bars indicate the number of dissected females. **B**: The precise number of parasites from 50 females of both groups was measured by Q-PCR 6 days after the second bloodmeal.

(i) 9 days before infection: *Phlebotomus duboscqi* females, previously fed either on chickens or mice, were given an infective bloodmeal nine days later and then dissected on days 2 and 6 PI. Data from three independent experiments were pooled. In both groups of females (fed on chicken versus mouse) similar *Leishmania* development was observed (Figure 2): no significant differences were found in infection rates or intensities of infection on days 2 and 6 PI (day 2: χ^2^ = 0.89, P = 0.64; day 6: χ^2^ = 0.10, P = 0.76). On day 6 PI, all females from both groups were infected and a majority of them had high parasite loads (over 80% had heavy infections); *Leishmania* promastigotes colonized the stomodeal valve in almost all females (99%). Various promastigote forms, mainly leptomonads and metacyclic promastigotes were present in thoracic midgut.

(ii) 1 day after infection: Females infected with *Leishmania* promastigotes in 5% rabbit blood were fed one day later on chickens or mice. Data from three independent experiments were pooled. On days 2, 6, and 9 after the second bloodmeal, no significant differences were observed in *Leishmania* development: females fed on chickens or mice did not differ in infection rates (χ^2^ = 1.37, P = 0.24) or intensities of infection on any of the compared days PI (day 2: χ^2^ = 2.81, P = 0.42; day 6: χ^2^ = 0.19, P = 0.76; day 9 χ^2^ = 3.07, P = 0.38). On day 9 after the second bloodmeal, 50% of females from both groups were infected; the intensities of infection were high in most of them and promastigotes colonized the stomodeal valve in 100% of infected females (Figure 3A). Various promastigote forms including metacyclics were observed. Similarly, Q-PCR revealed no significant differences (KW-H_(1;100)_ = 1.03, P = 0.31) in total parasite numbers in sand fly midguts on day 9 after the second bloodmeal (Figure 3B).

(iii) 6 days after infection: Females infected by *Leishmania* were fed on chickens or mice six days after an infective meal containing 10% rabbit blood with promastigotes. Data from two independent experiments were pooled. Females from both groups were dissected on days 2 and 6 after the second bloodmeal, and no significant differences were observed between experimental groups (chicken vs. mouse) in infection rates (χ^2^ = 0.05, P = 0.82) or intensities of infection (day 2: χ^2^ = 5.72, P = 0.13; day 6: χ^2^ = 0.08, P = 0.96). *Leishmania* developed similarly in both female groups: infection rates were about 80 − 85% and parasite loads were high in a majority of infected females (50 − 80%) (Figure 4A). Promastigotes colonized the stomodeal valve from day 6 after the second bloodmeal in 100% of infected females. Leptomonad and metacyclic forms prevailed in thoracic midgut. Similarly, Q-PCR revealed no significant differences (KW-H_(1;100)_ = 1.20, P = 0.27) in total parasite numbers in sand fly midguts on day 6 after the second bloodmeal (Figure 4B).

### Sand fly susceptibility to *L. major* infection during the first and second bloodmeals

**Figure 5:**
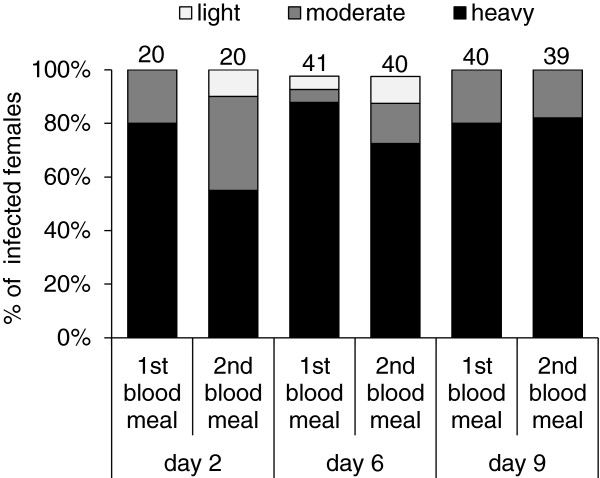
**Susceptibility to *L. major *infection during the first and second bloodmeal.***P. duboscqi* females were infected (10^6^ promastigotes/ml) during the first or second bloodmeal. Infection rates and intensities of infection were evaluated microscopically in sand fly midguts on days 2, 6, and 9 PI, and infections were classified into three categories: light (< 100 parasites/gut), moderate (100–1000 parasites/gut), or heavy (> 1000 parasites/gut). Numbers above the bars indicate the number of dissected females.

*Leishmania* development in females infected by rabbit blood with promastigotes during the first or second bloodmeal was compared on days 2, 6, and 9 PI. Data from two independent experiments were pooled. No significant differences were observed between experimental groups (1^st^ vs. 2^nd^ bloodmeal) in infection rates (χ^2^ = 0.0002, P = 0.99) or intensities of infection for any of the compared days PI (day 2: χ^2^ = 3.74, P = 0.15; day 6: χ^2^ = 3.41, P = 0.33). In late-stage infections, on days 6 and 9 PI, *P. duboscqi* females of both groups showed very high infection rates (almost 100%), high parasite loads (about 80% of heavy infections with metacyclic forms present in thoracic midgut) and a majority of females had the stomodeal valve colonized by promastigotes (Figure 5).

### The effect of blood concentration (5% vs. 100%) on parasite establishment in sand fly midguts

In experiments using *Leishmania* promastigotes in 5% rabbit blood, infection rates and parasite loads of infected females after the second bloodmeal were lower in comparison with females infected by feeding on 100% blood. Therefore, we decided to test if the infection rates and parasite loads differ already in the early stage of infection (day 1 and 2), thus before the time of the second feeding (as described in a previous experiment).

**Figure 6:**
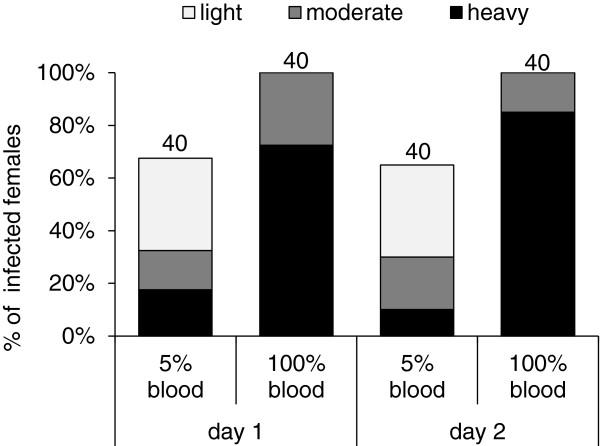
**The effect of 5% or 100% blood in infective bloodmeals on parasite establishment in the sand fly midgut.***P. duboscqi* females were infected (10^6^ promastigotes/ml) by feeding on 5% or 100% blood. Sand fly midguts of infected females were investigated microscopically on days 1 and 2 PI, and intensities of infections were classified into three categories: light (< 100 parasites/gut), moderate (100–1000 parasites/gut), or heavy (> 1000 parasites/gut). Numbers above the bars indicate the number of dissected females.

Two groups of females were infected by feeding on 5% or 100% rabbit blood with promastigotes and were dissected on days 1 and 2 PI. Data from two independent experiments were pooled and significant differences in infection rates (χ^2^ = 32.48, P < 0.001) and intensities of infection in both of compared days PI (day 1: χ^2^ = 41.92, P < 0.001; day 2: χ^2^ = 51.97, P < 0.001) were observed between the groups (5% vs. 100% blood). While only 65% of females fed on 5% blood with promastigotes were infected and the intensities of their infection were usually light or moderate, females fed on 100% blood were all infected and the intensities of their infections were higher (Figure 6).

### Differences in digestion of 10% and 100% blood

**Figure 7:**
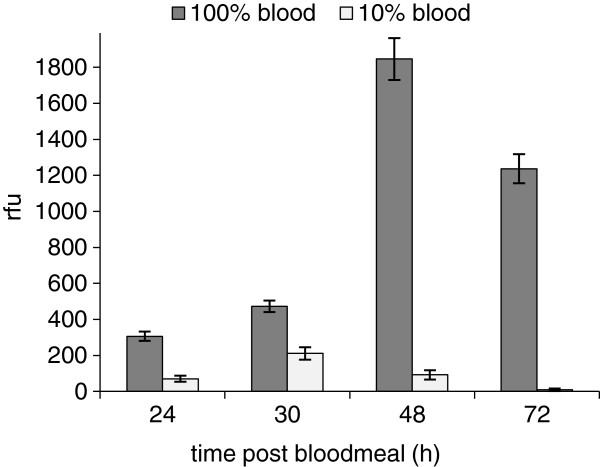
**Differences in digestion of 10% and 100% blood.** Trypsin activity was measured at 24, 30, 48, and 72 hours PBM in the homogenates of ten midguts of females fed on diluted (10%) or undiluted (100%) blood.

In experiments using promastigotes in 10% rabbit blood, infection rates and parasite loads of infected females after the second bloodmeal were slightly lower in comparison with females infected by feeding on 100% blood. Therefore, to explain this observation we decided to examine course of trypsin activity and the time of defecation after feeding on diluted or undiluted blood. Data from two independent experiments were pooled. Midgut trypsin activity in females fed on 10% blood was considerably lower and peaked earlier (Figure 7). The highest values were measured at 30 hours PBM, and by 72 hours PBM was almost zero. In contrast, midgut trypsin activity in females fed on 100% blood was the highest at 48 hours PMB and at 72 hours PMB was still relatively high. The maximum trypsin activity in females fed on 100% blood was ten times higher than those fed on 10% blood (Figure 7).

In addition, we compared the time of defecation: females fed on 10% blood defecated one or two days earlier compared to those fed on 100% blood. Females fed on full blood defecated on days 4 – 5 post bloodmeal while those fed on 10% blood defecated on day 3 post bloodmeal. Consequently, this provides considerably less time for *Leishmania* to escape from the peritrophic matrix and to establish an infection within the sand fly midgut.

## Discussion

Bloodmeals from different animal sources has been reported to affect the digestion, reproductive potential of females and development of *Leishmania* parasites in the midgut [3-7,15,23]. In this study, the effect of chicken blood on digestion, oocyte development and *Leishmania* infection within the sand fly gut was evaluated.

According to Sant’Anna et al. [17], chicken blood has less than half the total protein of rabbit blood, but the midgut protein content of fully engorged *L. longipalpis* females fed on rabbit blood was only slightly lower than that of females fed on chicken blood. Clearly, *L. longipalpis* females were able to partially compensate for the lower protein content in the avian blood source through efficient prediuresis [17]. In the experiments presented here *P. duboscqi* females fed on chickens had half the midgut protein content compared to those fed on rabbits, which corresponds to the concentrations measured in chicken and rabbit blood. As prediuresis has also repeatedly been described in *P. duboscqi* females [24,25], it seems that this species is not able to concentrate avian blood more than rabbit blood.

The lower protein content in the avian blood source influenced the midgut trypsin activity and oocyte development of *P. duboscqi*. Females fed on chickens had significantly lower trypsin activity in the midgut (18, 24, 30, and 72 hours PBM) and slower oocyte development (data not shown) in comparison with females fed on rabbits. These results are consistent with studies on the dependence of enzymatic activity on bloodmeal protein content in mosquitoes, where the proteolytic activity is activated by ingested proteins and that rate of proteolytic activity correlates with protein concentration in the bloodmeal [8,9].

In sand flies, proteins from the bloodmeal are digested for 48–96 hours [26-28], and it is during this time when *Leishmania* parasites encounter sand fly digestive enzymes. Some authors have shown that the digestion of blood from some hosts may adversely affect the development of *Leishmania*[14,15,23]. On the other hand, *Leishmania* was shown to modulate trypsin secretion of the sand fly vector to its own benefit. This effect has been described in the New World (*L. longipalpis* and *L. mexicana*) [29] as well as in the Old World parasite-vector pairs (*P. pernicious* and *L. infantum*) [30].

According to Schlein et al. [15] and Schlein and Jacobson [23] digestion of avian blood is harmful to *Leishmania* parasites within the sand fly midgut. They fed *P. papatasi* females on turkeys or chickens either before or after an infective meal containing rabbit blood with *Leishmania* promastigotes and in both experimental schemes described a reduction of *Leishmania* infection [15,23]. In contrast, chicken blood did not reduce the infection of *L. braziliensis*, *L. amazonensis* and *L. mexicana* in the New World sand fly species *L. longipalpis* and *L. migonei*[16,17]. Although Nieves and Pimenta [16] noted a slightly lower percentage of infected females after feeding an amastigote-infected chicken bloodmeal compared to females infected via rodent blood (*Cercomys* sp.), infections were not eliminated, and *L. braziliensis* and *L. amazonensis* established midgut infections. Sant’Anna et al. [17] also did not detect any negative effect of avian blood on *L. mexicana* infection in the midgut of *L. longipalpis*; on the contrary, in females infected via chicken blood they reported a trend towards higher infection rates and higher parasite loads in comparison with controls fed on infective rabbit blood [17]. While Sant’Anna et al. [17] used amastigote-initiated infections, in our study the infections were promastigote-initiated.

In the present work the effect of avian blood on the development of *L. major* in *P. duboscqi* was studied using light microscopy and Q-PCR in several experiment schemes where sand fly females were fed on chickens or mice either before or after infection. No significant differences were observed in any of these experiments and we can conclude that digestion of avian blood is not harmful to *L. major* development either before or after infection. The differences between our and Schlein’s results cannot be explained by different techniques or parasite vector pairs. We used the same *Leishmania* strain (LRC-L137), and *P. duboscqi* is the sister species of *P. papatasi* within the subgenus *Phlebotomus*, both being natural vectors of *L. major*[31,32].

*Phlebotomus duboscqi* females infected using the method of Schlein et al. [15] (infection by promastigotes in 5% rabbit blood and one day later fed on avian blood) had a relatively low (about 60%) infection rate in both groups, regardless of whether fed on chickens or mice. However, Schlein et al. [15] tested only the group fed on turkeys before or after infection and did not include any control group fed on a mammalian host. Therefore, their conclusions may have been influenced by the absence of appropriate controls. To confirm this assumption, we studied the effect of diluted blood on *Leishmania* development in the early stage of infection within the sand fly midgut. While females infected via 100% blood were all infected with high intensities of infection, females fed on 5% blood with promastigotes were infected in only 65% and parasite loads were light or moderate. This experiment revealed that diluted blood in infective meal leads to significantly lower infection rates and parasite loads, probably as a consequence of faster digestion. The peritrophic matrix of *P. duboscqi* females fed on full blood matures in about 12 hours PBM, and its disintegration started only at the third day PBM [33] and females defecated on days 4 – 5 PBM. On the other hand, in females infected via diluted blood *Leishmania* promastigotes have a very limited time to escape the peritrophic matrix and establish an infection within the midgut.

To complete the study on the influence of avian blood on *Leishmania* development we considered the effect of number of feedings and age of females on parasite development within the sand flies. Such effects have previously been described in other bloodsucking arthropods: tsetse flies (*Glossina* spp.) given trypanosomes in their first bloodmeal were found to be more susceptible to infection compared to flies given trypanosomes in a later bloodmeal [34]. More recent studies by Walshe et al. [35] and Kubi et al. [36] showed that it is rather the age (hours after eclosion) of the flies when they take the first infective bloodmeal or nutritional stress that determines the susceptibility to infection. It seems that a higher susceptibility to infection is caused by the physiological immaturity and imperfect immune response of teneral (newly emergent and unfed) tsetse flies [36]. Based on this knowledge, we decided to test the susceptibility of *P. duboscqi* to *L. major* infection during the first or the second bloodmeal; however, no differences between these two experimental groups of females were observed. This finding corresponds with the lack of any information regarding significant differences in infection rate after the first or the second blood feeding in Nematocera. Such a contrast between brachyceran and nematoceran flies (tsetse and sand flies, respectively) could be explained by differences in bloodmeal digestion mode and the type of peritrophic matrix (PM). Sand flies as well as mosquitoes and other nematoceran haematophagous insects have discontinuous bloodmeal digestion and form PM type 1, while tsetse flies digest blood continuously and form PM type 2 (reviewed by [37]).

## Conclusions

*Phlebotomus duboscqi* females fed on chicken had lower trypsin activity and slower oocyte development in comparison to those fed on mouse. Importantly, various experiments showed that the feeding of *Phlebotomus* sand flies on avian blood is not harmful to *Leishmania* development within their midgut. These experiments indicated that the reduction in *Leishmania* infection reported by Schlein et al. [15] and Schlein and Jacobson [23] was probably not caused by the inclusion of avian blood but by the experimental scheme using diluted blood.

In addition, the susceptibility of *P. duboscqi* females to *L. major* infection is equal during the first or the second bloodmeal; the number of feedings or female age did not affect the development of *Leishmania*.

## Competing interests

The authors declare that they have no competing interests.

## Authors’ contributions

KP performed the experimental work, analysed the data and wrote the manuscript. JV performed and analysed Q-PCR data, performed statistical analysis. PV designed the study, contributed to interpretation and wrote the manuscript. All authors read and approved the final version of the manuscript.
